# A data-driven, knowledge-based approach to biomarker discovery: application to circulating microRNA markers of colorectal cancer prognosis

**DOI:** 10.1038/s41540-018-0056-1

**Published:** 2018-06-01

**Authors:** Fatemeh Vafaee, Connie Diakos, Michaela B. Kirschner, Glen Reid, Michael Z. Michael, Lisa G. Horvath, Hamid Alinejad-Rokny, Zhangkai Jason Cheng, Zdenka Kuncic, Stephen Clarke

**Affiliations:** 1 School of Biotechnology and Biomolecular Sciences, University of New South Wales, Sydney, NSW 2033 Australia; 2 Kolling Institute of Medical Research, University of Sydney, Royal North Shore Hospital, Reserve Road, St Leonards, NSW 2065 Australia; 3 Asbestos Diseases Research Institute, Hospital Road, Concord, NSW 2139 Australia; 4 Sydney Medical School, University of Sydney, Sydney, NSW 2050 Australia; 5 Flinders Centre for Innovation in Cancer, Flinders Medical Centre, Flinders University, Adelaide, SA 5042 Australia; 6 Chris O’Brien Lifehouse, Missenden Road, Camperdown, NSW 2050 Australia; 7 Royal Prince Alfred Hospital, Camperdown, NSW 2050 Australia; 8 University of New South Wales, Sydney, NSW 2052 Australia; 9 Charles Perkins Centre, University of Sydney, Sydney, NSW 2006 Australia; 10 School of Physics, University of Sydney, Sydney, NSW 2006 Australia

## Abstract

Recent advances in high-throughput technologies have provided an unprecedented opportunity to identify molecular markers of disease processes. This plethora of complex-omics data has simultaneously complicated the problem of extracting meaningful molecular signatures and opened up new opportunities for more sophisticated integrative and holistic approaches. In this era, effective integration of *data-driven* and *knowledge-based* approaches for biomarker identification has been recognised as key to improving the identification of high-performance biomarkers, and necessary for translational applications. Here, we have evaluated the role of circulating microRNA as a means of predicting the prognosis of patients with colorectal cancer, which is the second leading cause of cancer-related death worldwide. We have developed a multi-objective optimisation method that effectively integrates a data-driven approach with the knowledge obtained from the microRNA-mediated regulatory network to identify robust plasma microRNA signatures which are reliable in terms of predictive power as well as functional relevance. The proposed multi-objective framework has the capacity to adjust for conflicting biomarker objectives and to incorporate heterogeneous information facilitating systems approaches to biomarker discovery. We have found a prognostic signature of colorectal cancer comprising 11 circulating microRNAs. The identified signature predicts the patients’ survival outcome and targets pathways underlying colorectal cancer progression. The altered expression of the identified microRNAs was confirmed in an independent public data set of plasma samples of patients in early stage vs advanced colorectal cancer. Furthermore, the generality of the proposed method was demonstrated across three publicly available miRNA data sets associated with biomarker studies in other diseases.

## MiRNA biomarker discovery: a network-based, knowledge-driven approach

The identification of robust and reproducible molecular markers is one of the biggest challenges in personalised cancer medicine. The increasing use of systems biology approaches has prompted researchers to integrate heterogeneous data into existing knowledge bases whose incorporation into the biomarker discovery workflow may adjust for data heterogeneity and limitation, and offer more precise, robust and consistent biomarkers. In this study, we have sought to determine network-based miRNA biomarker signatures from the plasma of colorectal cancer patients that hold prognostic utility. We performed miRNA profiling and then constructed an miRNA-mediated gene regulatory network and developed a multi-objective optimisation-based computational framework to identify miRNA biomarkers using both the miRNA expression profile and knowledge from this miRNA-mediated regulatory network. We have demonstrated the ability of the proposed approach in identifying robust, accurate and reproducible biomarkers.

## Introduction

The identification of robust and reproducible molecular markers is one of the biggest challenges in personalised cancer medicine. The complexity and heterogeneity of cancer, noise and nonlinearities in high-throughput data, and relatively small sample sizes can all contribute to the observed inconsistencies across different biomarkers reported for identical clinical conditions. However, the increasing use of systems biology approaches has prompted researchers to integrate heterogeneous data into existing knowledge bases in order to facilitate the system-level understanding of disease. Now, incorporating such knowledge bases into the biomarker discovery workflow may adjust for data heterogeneity and limitation, and offer more precise, robust and consistent biomarkers.^1^

Colorectal cancer (CRC) is the second leading cause of cancer-related mortality both in Australia^2^ and worldwide,^3^ and in Australia, is the second-most prevalent cancer in both men and women.^2^ While survival rates have increased over the past 30 years with the introduction of screening programmes and new systemic treatment agents, the 5-year relative survival from CRC remains only 68%.^2^ Of those patients who undergo curative surgery for CRC, one in three will experience disease recurrence.^4^ For patients with metastatic disease, 5-year survival is only around 13%.^5^ An important challenge, therefore, is identifying those patients who have undergone curative resection who are at higher risk of recurrence and selecting those likely to derive benefit from adjuvant chemotherapy. Similarly, for patients with metastatic disease, early identification of those who are likely to develop more severe toxicities or derive little or no response from what can be expensive cytotoxic and targeted agents would allow for the selection of alternate, better tolerated therapies; tailored doses of the same agents; or the use of prophylactic supportive therapies, such as antibiotics or growth factors. These limitations highlight the need for novel biomarkers that facilitate the early identification of patients with poor prognosis.

Currently, performance status and cancer stage are the main indicators for treatment selection and survival prognostication. There is now a crucial need for the personalisation of treatment using molecular biomarkers, in conjunction with baseline clinical and laboratory variables. Blood-based biomarkers are particularly attractive given that blood is a readily available, minimally invasively obtained medium that allows for simple, inexpensive and repeated sampling.

MicroRNAs (miRNAs) are small (19- to 25-nucleotide) noncoding RNA molecules that regulate gene expression at the translational level. They are involved in a number of biological processes, including human cancers, where they are differentially expressed.^6^ MiRNAs have been shown to have roles as tumour suppressor genes and oncogenes, and their diagnostic, prognostic, predictive and therapeutic implications are now being explored. Both plasma and serum are stable sources of circulating miRNAs^7,8^ and both are suitable for investigations of miRNAs as blood-based biomarkers.^7^

Several studies on colorectal tumour tissue or cell lines have been performed which have sought miRNAs for use as prognostic or predictive biomarkers, and those involved in biological processes such as tumorigenesis and metastasis.^9,10^ Plasma-derived miRNAs have been mostly used as diagnostic biomarkers in CRC patients.^11,12^ However, while a few studies to date have examined the utility of circulating miRNAs as *prognostic* CRC biomarkers,^13–16^ the reported miRNAs do not overlap between studies.

Predicting patient clinical outcomes via molecular expression information has traditionally focused on the study of individual molecules (i.e., differential expression analysis). This approach, however, does not adequately take into account the informational complexity underpinning many clinical states. The over-reliance on such hypothesis-driven, reductionist approaches to biomarker discovery, despite the valuable achievements so far, may limit the translation of fundamental research into new clinical applications due to their limited ability to unravel the multivariate and combinatorial characteristics of cellular networks implicated in multi-factorial diseases such as cancer.^17^

Instead, *systems*-based biomarker discovery approaches may more accurately reflect the underlying biology than traditional reductionist approaches. In this context, biomarkers, as indicators of a clinical state, are computationally derived from networks of interacting molecular entities and incorporate measurements from the expression of molecules with the information on clinically meaningful biological interactions.^17^

In recent years, network-based approaches of gene expression analysis have grown in popularity for their capacity to explain emergent properties such as biological heterogeneity, modularity or phenotypic variability.^18^ It has been frequently shown that molecular networks (e.g., protein−protein interaction, gene regulatory and signalling networks) are sources for identifying powerful biomarkers; network-based biomarkers can capture changes in downstream effectors and in many cases are more useful for prediction compared to any individual gene.^19–23^ Several approaches exist involving the utilisation of networks of molecular interactions in gene expression signature modelling.^20,24–26^ Nonetheless, the advantage of network-based approaches has rarely been applied to miRNA biomarkers, possibly because miRNA networks are not prevalent and readily available as opposed to gene or protein interaction networks.

It is well understood that miRNAs cooperate to achieve gene regulation and that each miRNA has the potential to target a large number of genes.^27^ Our increasing knowledge of the miRNA-mediated regulatory network has underlined the importance of miRNA control over tumour cell biology. miRNAs associated with patient outcome have been found to be oncogenic or tumour suppressive, affecting multiple cancer-associated pathways by targeting oncogenes or tumour suppressor genes.^6^ Overall, the miRNA-mediated gene regulatory network carries key information on the functional role of miRNAs in cancer whose utilisation in miRNA expression signature modelling may lead to the identification of biologically relevant markers when miRNAs are released from cancer cells, or linked to systemic processes.

In this study, we have sought to determine network-based miRNA biomarker signatures from the plasma of CRC patients that hold prognostic utility. To this end, we performed miRNA profiling and then constructed an miRNA-mediated gene regulatory network, and developed an innovative *multi-objective optimisation*-based computational framework to identify miRNA biomarkers using both the miRNA expression profile and information from this miRNA-mediated regulatory network.

## Methods

### Patient selection, blood collection and preparation of plasma

Patients with a histologically confirmed diagnosis of locally advanced or metastatic CRC receiving adjuvant or palliative chemotherapy respectively attending the medical oncology outpatients’ clinics at Concord and Royal Prince Alfred Hospitals in Sydney, Australia, were eligible for inclusion. Patients were required to have good performance status (ECOG 0−2), and adequate organ function. Patients were excluded if they had prior chemotherapy for metastatic CRC or completed adjuvant chemotherapy within the past 6 months. This study was performed in accordance with relevant guidelines and regulations and with the approval of the individual ethics committees of the institutions where the patients were being treated.

Plasma samples were taken prior to commencing chemotherapy. Blood was collected by routine venepuncture in 10 ml Vacutainer Plus K_3_EDTA tubes (BD Biosciences). Tubes were inverted ten times immediately after collection, and were centrifuged at 2500 × *g* for 20 min at room temperature within 30 min of collection. Plasma was stored at −80°C until further processing.

### RNA isolation, quality control and OpenArray analysis

Total RNA was isolated from plasma using the MirVana PARIS miRNA isolation kit (Ambion/Applied Biosystems, Foster City, CA) according to a modified protocol.^28^ Isolated plasma samples were assessed for haemolysis by examination of free haemoglobin and miR-16 levels, the latter being an miRNA found in red blood cells. Quantification of free haemoglobin was performed as described previously^28^ on an Implen Nanophotometer (Implen GmbH, Munich, Germany), and miR-16 levels were quantified by real-time RT-qPCR. Quantification details are provided in the Supplementary file 1, Section 1.1. Samples deemed haemolysed were excluded from further analysis.

Global profiling of miRNAs in the plasma samples was carried out using the OpenArray platform (Applied Biosystems), according to the manufacturer’s instructions. The entire RT reaction was used for pre-amplification carried out on a ViiA 7 instrument (Applied Biosystems). The resultant cDNA was combined with the OpenArray real-time PCR Master Mix and loaded onto the OpenArray miRNA panel plates (Applied Biosystems) using the AccuFill autoloader. The loaded plates were run on the BioTrove OpenArray real-time PCR instrument (Flinders Medical Centre, SA) and run according to the default protocol for reaction conditions. See Supplementary file 1, Section 1.2 for details.

### Statistical data preprocessing

The pre-processing of miRNA cycle quantification (Cq) values from quantitative RT-qPCR assays were performed using MATLAB 2014b, Bioinformatics Toolbox and Statistics Toolbox. The preprocessing workflow includes quality assessment, normalisation and filtering. The chosen parameters are justified in Supplementary file 1, Section 1.3. QC plots for non-detects and Cq distributions were used to examine the quality of the data and deviated trends. Quantile normalisation was used to adjust for technical variability across multiple samples. MiRNAs that are missing in >50% of samples were excluded to acquire acceptable distribution of non-detects for down-stream analysis. Missing data was imputed using the nearest-neighbour method (KNNimpute), shown to be one of the most sensitive and robust methods for missing value estimation in expression data.^29^ Patients were dichotomised to long vs short survival using a 2-year cut-off point. To adjust for unbalanced class distribution, the under-represented class (i.e., short survival) was doubled using SMOTE: Synthetic Minority Oversampling Technique^30^ as implemented by the R ‘DMwR’ package. Oversampling was only used in the model selection phase to highlight performance differences across compared classifiers. Original data was used for the identification of the final miRNA signature reported in this study. Given that the data was not normally distributed, differential expression analyses were conducted using non-parametric approaches, namely two-sample Kolmogorov−Smirnov (KS) and Wilcoxon tests for the null hypothesis that the miRNA Cq values in short vs long survival patients are from the same continuous distribution.

### Biomarker discovery

#### Biomarker identification as an optimisation problem

Identification of a prognostic molecular expression signature can be thought of as a problem of finding a set of molecules (e.g., miRNAs) whose expression profile best stratifies patients into the groups of interest—i.e., shorter vs longer survival. This can be modelled as an optimisation problem that is defined as finding a solution, out of all possible solutions, that minimise/maximise an objective function. An optimisation problem is typically formulated as $\mathop {{\min }}\nolimits_x f(x)$, s.t. $x \in X$, where *X* is the set of all possible solutions and *f*:*x*→ℝ is an objective function that maps any feasible solution onto a real number evaluating the ‘goodness’ of the solution instance. By convention, the standard form defines a minimisation problem. A maximisation problem can be treated by negating/inversing the objective function. In this study, we used a popular and powerful class of optimisation algorithms known as evolutionary algorithms (EAs).^31^ EAs are generic population-based metaheuristic optimisation algorithms whose mechanisms are inspired by biological evolution. An EA procedure begins with a population of solutions usually generated at random. It then iteratively updates the current population to generate a new population by the use of four main operators, namely selection, crossover, mutation and elite-preservation. The operation stops when one or more pre-specified termination criteria are met (e.g., the optimum is found, the population is converged, or a pre-specified number of generations is passed).

An EA relies on the specification of (1) solution instance, and (2) objective function (usually referred to as the fitness function). Here, a solution instance encodes a set of miRNAs selected out of all *N* miRNAs under study and is represented by a binary string of length $l = \left| N \right|,$ where each bit in the string corresponds to a particular miRNA, *m*_*i*_ whose value ‘1’ or ‘0’ encodes the inclusion or exclusion of *m*_*i*_, respectively. Each solution can be thought as a potential biomarker and the optimisation algorithm searches for a set of miRNAs whose expression profile best *classifies* patients into groups with shorter vs longer survival. Therefore, to evaluate each solution, the expression values of the corresponding miRNAs are fed into *a classifier* which is an algorithm or a function that maps these expression values (known as features) to the binary space of long or short survival. The classification error rate is then considered as the fitness function and the EA is set to find a solution with minimal misclassification rate.

#### Construction of miRNA-mediated gene regulatory network

We have developed an algorithm that constructs a network of miRNA-mediated regulatory cascades and used this network to discover miRNA signatures. In a mathematical formulation, a network or a graph consists of a set of nodes *V* and a set of edges *E* between nodes. Here, a node is an miRNA or a gene and an edge is a directed association representing the regulation of a target gene (TG) by the source nodes that is either an miRNA or a transcription factor (TF). Human miRNA targets were retrieved from publicly available data sets of experimentally validated and predicted data sets using *multiMiR*^32^—updated on 12/22/2016. MultiMiR is an miRNA-target interaction R package and database that compiles nearly 50 million records in human and mouse from 11 different databases: validated targets were collected from miRecords,^33^ miRTarBase,^34^ and TarBase^35^ and predictions from DIANA-microT-CDS,^36^ ElMMo,^37^ MicroCosm, miRanda,^38^ miRDB,^39^ PicTar, PITA,^40^ and TargetScan.^41^ Targets of miRNAs under study were included in the network if experimentally validated or predicted by at least two databases. Additionally, a gene regulatory network—i.e., a collection of validated TF−TG interactions—was obtained from the ORTI database,^42^ an open-access comprehensive repository of regulatory interactions that compiles mammalian TFs and their associated TGs from publicly available databases of TF−TG interactions, namely HTRI,^43^ TFactS,^44^ TRED,^45^ TRRD,^46^ PAZAR,^47^ and NFI-Regulome,^48^ and the literature. The miRNA-mediated regulatory network was then constructed using an iterative process as outlined below:

Starting from an empty network, the set of miRNA-target interactions for each miRNA under study were first added to the network. The miRNA targets may comprise TFs that can in turn target other genes and pass on the regulation to the second level. Those TF−TG interactions were then added to the network. Similarly, the newly added TGs (i.e., the targets of the targets of the miRNAs) may contain TFs that extend the regulation cascade to deeper levels. This process continues until ‘convergence’, i.e., when no new TF−TG interaction can be added to the network, meaning that all TFs and TGs reachable from the initial miRNAs have already been traversed and added to the network. The pseudocode of an efficient recursive implementation of the proposed algorithm is shown in Supplementary file 1, Section 1.4.

#### Annotation of the CRC-related genes on the network

The miRNA-mediated regulatory network can be used to identify miRNAs which target, either directly or indirectly, genes functionally associated with CRC, and thus have the potential to play a role in the cellular mechanisms underlying CRC pathogenesis. This requires the annotation of the network genes according to their association with CRC. We used the MalaCards human disease database,^49^ which is an integrated compendium of annotated diseases mined from multiple data sources. MalaCards provides the list of genes affiliated with a queried disease accompanied with a prioritising algorithm to rank the gene list. It distinguishes ‘*elite*’ genes as those likely to be associated with causing the disease, since their gene–disease associations are supported by manually curated and trustworthy sources. The relevance of the MalaCards retrieved genes to CRC were ranked into two levels—rank ‘1’ for elite genes and rank ‘2’ for the rest of CRC associated genes. These genes were then annotated with their ranks on the miRNA-mediated regulatory network.

#### Network-based CRC functional relevance score

An miRNA can target multiple CRC-related genes either directly or indirectly. The probability of the mechanistic involvement of an miRNA in CRC increases if the miRNA targets *more* CRC genes in *a shorter* distance within the network. We aggregated these measurements into a scoring function to quantify the functional relevance (FR) of each miRNA to CRC pathogenesis for the subsequent biomarker modelling. Equation (1) shows the FR formulation, where *m*_*i*_ is an miRNA in the miRNA-mediated regulatory network, *TG* = {*g*_*k*_} is the set of all CRC TGs reachable from *m*_*i*_ on the network, *d*(*m*_*i*_, *g*_*k*_) is the shortest distance from *m*_*i*_ to *g*_*k*_ on the network computed using the Bellman–Ford algorithm,^50^ and *r*_*gk*_ is the CRC rank assigned to TG *g*_*k*_. *ε* is a small constant (i.e., 10E-3) to avoid *FR* = 0 and ‘division by zero’ in subsequent analyses.

1 $$FR\left( {m_i} \right) = \varepsilon + \mathop {\sum }\limits_{g_k \in TG} \exp \left( { - \left[ {d\left( {m_i,g_k} \right) + r_{g_k}} \right]} \right).$$

**Fig. 1:**
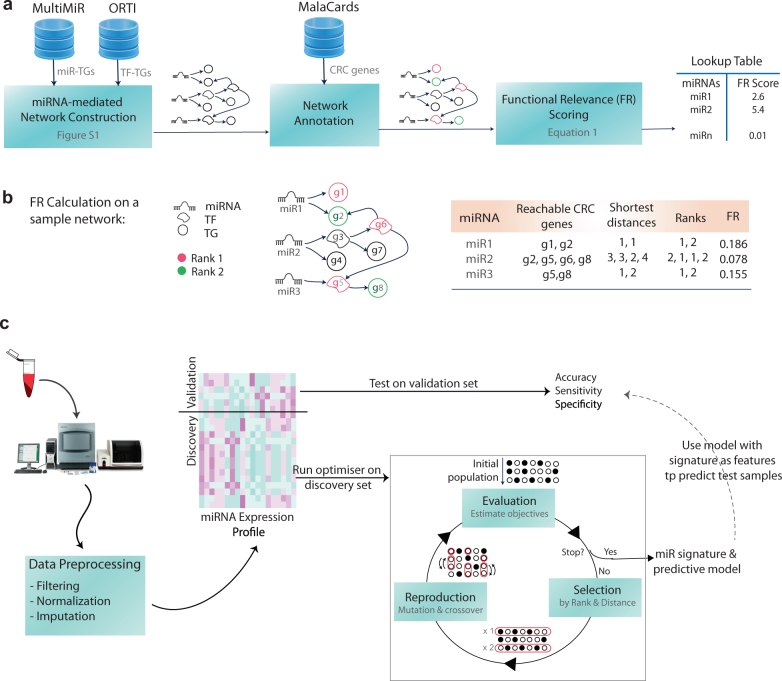
Outline of the method. **a** The construction steps of the miRNA-mediated regulatory network: (1) miRNA target genes (TGs) that are either validated experimentally or predicted by two different data sets were retrieved using multiMiR which is an R package providing access to 11 publicly available data sets. Transcription factor (TF) targets were retrieved from ORTI database which compiles validated mammalian TF-TG interactions from six public data sets as well as the literature. The miRNA-mediated regulatory network was constructed using a recursive algorithm described in Supplementary Figure S3. (2) The network was then annotated using 339 CRC-associated genes identified by MalaCards; 35 ‘elite’ genes with strong causal associations with CRC progression were ranked ‘1’ and the rest of CRC genes were ranked ‘2’. (3) Using the annotated network, a functional relevance (FR) score was calculated for each miRNA (using Eq. (1)) and a look up table was returned to be used in the subsequent biomarker discovery. **b** FR calculation on an example network. **c** Schematic view of the proposed multi-objective optimisation-based biomarker discovery workflow: The pre-processed samples were partitioned to validation and discovery sets using fivefold cross-validation. The multi-objective optimiser was run on discovery set where objectives are prediction errors and averaged FR scores of the population of putative signatures. Optimal miRNA signatures (i.e., Pareto front solutions) and their corresponding predictive models were then used to classify test samples and the performance measures were reported. The whole process repeated for 50 times to account for random partitioning of samples and the average performance measures were reported (Fig. 3)

According to this formulation, the farther the distance (or the higher the rank), the higher the magnitude of the exponent, and thus, the smaller the increment of the aggregated FR score. Figure 1b exemplifies FR calculation on a schematic miRNA network.

#### Multi-objective optimisation: essentials

A multi-objective optimisation is an optimisation problem that involves multiple objective functions, formulated as: $\min f_1(x), \ldots ,f_k(x)$, where integer $k \ge 2$ is the number of objectives *x* is a solution instance in the solution space *X* and $f:x \to {\cal R}^k$ is an objective function that maps each solution instance into a vector of real-valued vector of objectives.

In non-trivial multi-objective optimisation problems where the objective functions are conflicting, no feasible solution that simultaneously minimises all objective functions typically exists. Therefore, attention is paid to *Pareto optimal* solutions, i.e., solutions that cannot be at least one of the other objectives. A feasible solution $x_1 \in X$ is said to (Pareto) dominate another solution $x_2 \in X$, if:

$$\begin{array}{l}f_i\left( {x_1} \right) \le f_i\left( {x_2} \right)\,\forall i \in \left\{ {1,2 \ldots ,k} \right\},\cr f_j\left( {x_1} \right) < f_j\left( {x_2} \right)\,\exists j \in \left\{ {1,2 \ldots ,k} \right\}.\end{array}$$

A solution $x_1 \in X$ is called Pareto optimal if it is not dominated by any other solution in the solution space.^51^ The set of all feasible non-dominated solutions in *X* is referred to as the *Pareto optimal* set, and the corresponding objective vectors are called the *Pareto front*. For many problems, the number of Pareto optimal solutions is enormous and a multi-objective optimiser is usually aimed at identifying a representative set of solutions which (1) lie on the Pareto front, and (2) are diverse enough to represent the entire range of the Pareto front.^52^

A popular approach to generate Pareto optimal solutions is to use EAs. The use of a population of solutions allows an EA to find multiple optimal solutions, thereby facilitating the solution of multi-objective optimisation problems. Furthermore, EAs have essential operators to converge towards a set of non-dominated points which are as close as possible to the Pareto-optimal front, and yet diverse among the objectives.^53^

Currently most evolutionary multi-objective optimisation algorithms apply Pareto-based ranking schemes. A standard example is the *Non-dominated Sorting Genetic Algorithm-II* (NSGA-II).^54^ NSGA-II sorts the population into various fronts such that the first front is a completely non-dominant set in the current population (rank 1 individuals), and the second front is only dominated by the individuals in the first front (rank-2 individuals) and this process continues until the entire population is ranked. In addition to the individuals’ ranks, another parameter called *crowding distance* is calculated for each individual. Crowding distance is a measure of how close an individual is to its neighbours. NSGA-II selects individuals based on the rank and the crowding distance.

#### Multi-objective optimisation in network-based miRNA biomarker discovery

We developed a bi-objective optimisation workflow to identify multiple miRNA biomarkers by simultaneously optimising for two objectives: (1) the *predictive power* and (2) *functional relevance*. We used NSGA-II^54^ to search for multiple sets of plasma miRNAs whose expression profiles can precisely predict patients’ survival outcome and, at the same time, target CRC pathways on the miRNA-mediated regulatory network. The predictive power was estimated as the minimal misclassification rate using a classifier, and the functional relevance for each putative biomarker was estimated by aggregating over FR scores of the corresponding biomarker miRNAs. In mathematical terms, let ${\cal X} = \left\{ {m_i} \right\},i = 1, \ldots ,n$ be the set of all $n$ miRNAs under study, and ${\cal X}_i \subseteq {\cal X}$ be a subset of miRNAs (i.e., a solution or a putative biomarker), the optimisation problem is then formulated as

$$\min \left\langle {Err\left( {{\cal X}_i} \right),\left. {1/\overline {FR} \left( {{\cal X}_i} \right)} \right)} \right\rangle {{\mathrm s.}{\mathrm t.}}\,{\cal X}_i \subseteq {\cal X},$$

where the biomarker functional relevance is computed by:

$$\overline {FR} \left( {{\cal X}_i} \right) = \mathop {\sum }\limits_{m_k \in {\cal X}_i} FR\left( {m_k} \right).$$

The functional relevance shall be maximised and thus inverted to adhere with the standard minimisation problem. $Err\left( {{\cal X}_i} \right)$ is the average of error rates (i.e., number of incorrectly classified samples divided by total number of samples) over multiple runs of fivefold cross validation using *X*_*i*_ expression profile as the classification feature set. Figure 1c illustrates the proposed biomarker discovery workflow. NSGA-II parameters were set as follows: population size was set to 100, (scattered) crossover and (uniform) mutation rates were set to 0.8 and 0.01, respectively. The maximum number of generations was set to 50. The solver stops after iterating for 50 generations or when the average change in the spread of the Pareto front is less than 1E-4. *Crowding distance* was used as the distance function and Pareto front population fraction was chosen to be 20%. The workflow was coded in MATLAB R2014b and R. MATLAB optimisation toolbox was used to implement NSGA-II. Codes are available at https://github.com/VafaeeLab/multiobj_miR_marker_discovery.

#### Significance assessment of identified biomarkers

The statistical significance of each biomarker/Pareto solution was assessed using permutation hypothesis testing. Accordingly, for each Pareto solution an equivalent random individual was generated which has an equal number of miRNAs, but randomly chosen from the pool of miRNAs under study. The objective vector of the random solution was then estimated and this process was repeated 1000 times to generate a null distribution of objective vectors. For ease of assessment, we replaced each objective vector with a scalar value by computing its Euclidean distance with the ideal optimum that is origin 〈0; 0〉. The nominal *p* value for each Pareto solution/biomarker was then calculated as the proportion of random samples whose distance to origin is closer than or equal to that of the Pareto solution.

### Validation of altered expression of identified miRNAs in an independent data set

The altered expression of the identified miRNAs was examined in an independent public data set of qPCR miRNA CRC patient plasma samples^55^ which employed TaqMan Array Human MicroRNA Cards Set v2.0A/B and profiled the expression of 667 miRNAs in 48 plasma samples that included patients with normal, polyps, adenoma, early-stage (stage I/II) and advanced (stage III/IV) cancer. We downloaded the raw data from NCBI GEO archive (accession no: GSE67075). For consistency, we followed the same statistical pre-processing pipeline that we used to analyse our own data set. We performed differential expression analysis using the two-sample Wilcoxon test as implemented by R ‘HTqPCR’ package to compare the early-stage vs advanced groups (8 samples per groups) and reported the *p* values of miRNAs of interest. The statistical significance of the proportion of identified miRNAs differentially expressed in the validation data set was assessed using the right-sided Fisher’s exact test (‘stat’ R package, ‘phyper’ function).

### Pathway overrepresentation analysis

We were interested to examine whether the identified miRNAs enrich pathways relevant to CRC progression noting that pathway information was not used to obtain miRNA FR scores. We used KEGG pathways retrieved from the Molecular Signatures Database (MSigDB)-V 6.0.^56^ Targets of the identified miRNAs were extracted from the miRNA regulatory network and underwent pathway enrichment analysis using the right-sided Fisher’s exact test whose *p* value for the null hypothesis is computed based on the hypergeometric distribution:

$$p{\mathrm{ = }}\frac{1}{{\left( {\begin{array}{*{20}{c}} N \cr n \end{array}} \right)}}\mathop {\sum }\limits_{i = k}^{i = n} \left( {\begin{array}{*{20}{c}} n \cr i \end{array}} \right)\left( {\begin{array}{*{20}{c}} {N - K} \cr {n - i} \end{array}} \right),$$

where *N* is the total number of annotated genes, *n* is the number of genes targeted by signature miRNAs, *K* is the total number of genes annotated by a pathway, and *k* is the number of TGs in the pathway; *p* values were adjusted for multiple hypothesis testing using FDR correction. The analysis was implemented in R using ‘stats’ packages.

## Results and discussion

### Patient characteristics and data preprocessing

The characteristics of patients included in this study are shown in Table 1 and detailed in Supplementary file 2. Plasma samples were profiled against 557 miRNAs whose Cq values are shown as a heatmap in Supplementary file 1, Figure S3. Names of miRNAs were standardised to miRBase-Version 21 using miRSystem;^57^ 12 miRNAs that were unavailable or dead were excluded. For reliable downstream analysis, miRNAs missing in > 50% of samples were filtered out, resulting in 150 miRNAs.

**Table 1:** Baseline patient characteristics

Characteristics	*n* = 75	Description
Gender (F/M)	30/45	F: Female, M: Male
Age	59 years	Average age at enrolment
Survival (mean ∓ std)	20.98 ∓ 11.67 months	Survival times for 53 patients have not been reported as they have been alive at the end of the follow-up and their prognostic status was considered as ‘long survival’.
Tumour site (C/R/RS)	45/24/6	C: Colon, R: Rectum, RS: Rectosigmoid
Chemotherapy regime	FOLFOX	All 75 patients received FOLFOX-based chemotherapy

### MiRNA-mediated gene regulatory network

Figure 1a depicts the workflow of *miRNA-mediated regulatory network* construction. The constructed network comprises 150 miRNAs under study, 591 TFs and 22,635 TGs with a total number of 170,617 interactions including both miRNA-TG and TF-TG interactions. The network flat file is provided in Supplementary file 3. Once the network was constructed, CRC-related genes/nodes on the network were marked and ranked. Overall, 339 genes including 35 elite genes were annotated and ranked. CRC-associated genes, including elite ones and data sources used by MalaCards to imply CRC associations, are listed in Supplementary file 4. Lastly, the functional relevance of each miRNA was scored based on the rank and distance of miRNA’s CRC-related targets on the network. The proposed functional relevance (FR) scoring function takes into account direct miRNA targets as well as distant targets; yet, the farthest a target is, the lower its contribution to the FR score. Figure 1b schematically illustrates the FR calculation on a sample miRNA network; the histogram of FR score distribution is shown in Supplementary file 1, Figure S4.

### Performance comparison of different classifiers

**Fig. 2:**
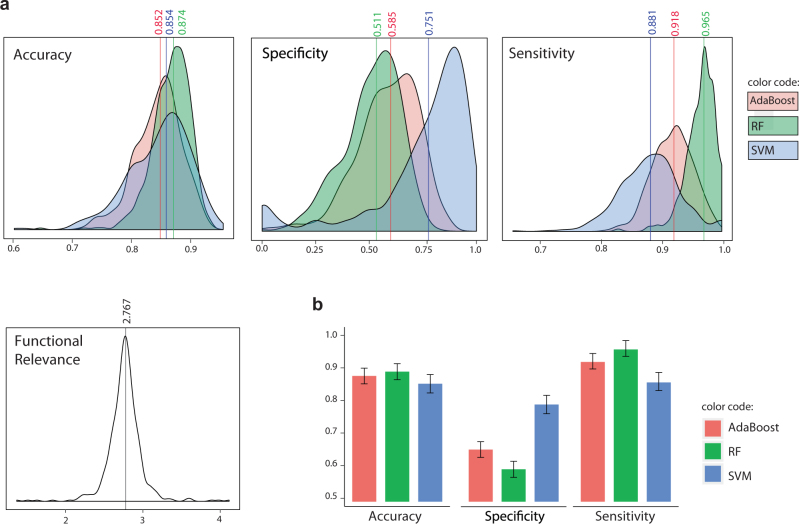
Performance comparison of different classifiers. The predictive performance of three different classifiers namely AdaBoost (with decision trees as weak learners), Random Forest (RF) and Support Vector Machine (SVM) were assessed. a Predictive features were selected randomly; the null distributions were set using 500 sets of randomly chosen miRNAs. The distributions of classifiers’ accuracy, specificity and sensitivity (with ‘long survival’ as positive class) as well as functional relevance scores were plotted. Mean values are marked on density plots. **b** The predictive features were the set of differentially expressed genes (KS test, *p* value < 0.05); error bars show standard deviations

An optimisation-based approach to biomarker discovery requires a choice of classifier to compute the fitness (e.g., misclassification rate) of solutions (i.e., putative signatures). We compared the performance of Support Vector Machine (SVM),^58^ Random Forest (RF)^59^ and AdaBoost^60^ with decision trees as weak learners as choices of classifiers. The expression profiles of differentially expressed miRNAs (i.e., *p* value < 0.05 using the KS test) were set as classifier features, which is a commonly used approach for feature selection in biomarker identification.^13–16,55^ The list of differentially expressed miRNAs is available in Supplementary file 5. We also measured the performance of different classifiers on a population of 500 randomly selected sets of features (i.e., miRNAs) providing the null distributions (Fig. 2a). Samples were divided into discovery and validation sets using fivefold cross-validation; cross-validation was then repeated ten times to account for random data splitting (total of 50 independent runs). In each run, classifiers were trained on the discovery sets and used to predict the corresponding validation samples. The predictive performance of the classifiers in terms of accuracy, sensitivity and specificity was estimated by averaging over 50 rounds of predictions; ‘long survival’ was considered as the ‘positive’ class implying that sensitivity is the classifier’s ability to correctly identify patients with long survival whereas the specificity represents the ability to correctly classify short survival. While all classifiers performed similarly in terms of total misclassification rates or accuracy (Fig. 2b), SVM significantly outperformed other classifiers on detecting the under-represented event of ‘short survival’ (i.e., higher specificity) and was thus chosen as the choice of classifier in the optimisation processes (Fig. 2b).

### Performance comparison with relevant approaches

To investigate the advantage of network-based multi-objective optimisation workflow proposed in this work, we compared the performance of resultant signatures with those achieved by a single-objective optimisation approach. In this latter approach, a genetic algorithm (GA), with similar experimental setup, was used to find sets of miRNAs with utility as a prognostic biomarker, by minimising the error rate in predicting patients’ survival status. Single-objective optimisation has previously been used for biomarker discovery in other contexts and has shown superior prediction performance as compared to conventional approaches. For instance, Liu et al.^61^ used GA combined with SVM classifier to identify biomarkers for tumour categorisation. As another example, Petricoin et al.^62^ reported the use of self-organising map coupled with GA to search through raw mass spectrometry data to identify a proteomic pattern discriminating ovarian cancer from non-cancer.

Optimisation-based approaches to biomarker discovery inherently select features through the search process. We also included into the comparison more classical models with the built-in feature selection capacity. Accordingly, we evaluated the *least absolute shrinkage and selection operator*^63^ (Lasso), a commonly used regression method that inherently performs variable selection by producing coefficients that are exactly 0. We used the ‘glmnet’ R package to fit the generalised linear regression model with Lasso; a lambda value that gives minimum mean cross-validated error was used for prediction and extraction of model coefficients. We also assessed RF and SVM with automatic feature selection. We used the ‘RRF’ R package to implement *guided regularised random forest* (guided RRF).^64^ The coefficients of regularisation were set to the normalised importance score of the variables as recommended in the RRF package.^65^ We also adopted the ‘penalizedSVM’ R-package that implements penalty functions for automatic feature selection in SVM classification.^66^ We chose the penalty function to be *Smoothly Clipped Absolute Deviation* (SCAD)^67^ due to its superior performance.^66^

**Fig. 3:**
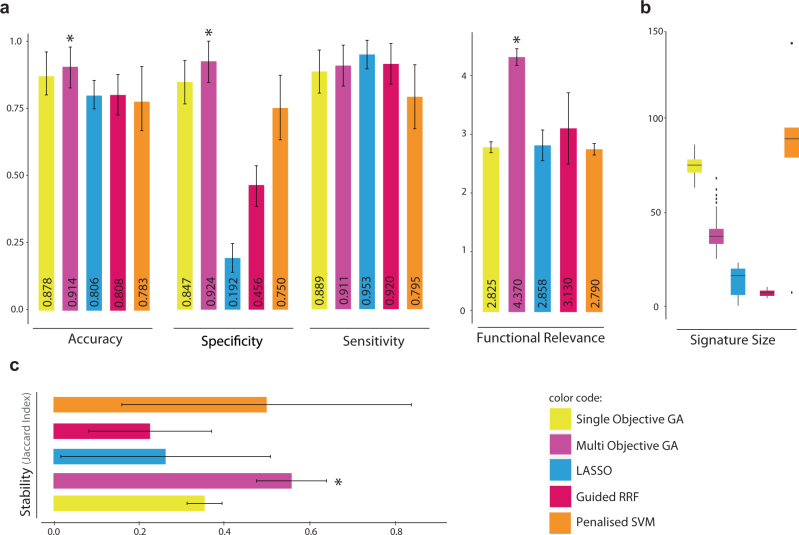
Performance comparison with relevant approaches with inherent feature selection. The performance of the proposed multi-objective optimiser was compared with relevant methods with inherent feature selection—i.e., single-objective optimiser, Lasso, guided RRF and penalised SVM. **a** The accuracy, specificity sensitivity and functional relevance score were averaged over 50 runs of sample partitioning using fivefold cross validation. **b** Sizes of the identified signatures or the number of features selected by each method over 50 independent runs were shown as box plots. **c** As a measure of signature stability, Jaccard Index was computed for all pairs of signatures identified by each of compared methods across 50 runs and the average values were reported. In all bar charts, error bars show standard deviations and multi-objective optimiser bars were marked by ‘*’ when the proposed method significantly outperforms others (Wilcoxon test *p* values < 0.001)

A similar pre-processing pipeline was followed for all compared algorithms. Again, samples were divided into discovery and validation sets using fivefold cross-validation and repeated ten times (50 independent runs). In each run, compared methods (i.e., multi-objective optimiser, single-objective optimiser, Lasso, guided RRF and penalised SVM) were trained on the discovery sets. The end-of-run models with the selected features were then used to predict validation samples and average accuracy, sensitivity and specificity were reported. The functional relevance scores of the identified signatures (i.e., selected features) were also averaged across 50 runs and reported to compare the biological implication of the identified biomarkers in CRC underlying mechanisms. As Fig. 3a shows, Lasso was unable to predict samples with ‘short survival’ and usually assigned all test samples to a single class of ‘long survival’. We therefore observed a very low specificity and high, but false, sensitivity with Lasso. Single- and multi-objective optimisers performed comparatively better than the other compared methods. Yet, multi-objective optimisation performed significantly better than single-objective optimisation on accuracy, specificity and functional relevance (Wilcoxon test *p* value < 0.001). We observed that single-objective optimisation overfitted to training data while multi-objective optimisation produced comparative performance on training and test sets and thus better generalised to independent data sets (Supplementary file 1, Figure S5). This demonstrates the advantage of using a data-independent knowledge-based approach in avoiding overfitting to data. Along the same lines, the multi-objective optimiser also controls for signature sizes (Fig. 3b). Large signatures usually produce excessively complex models overreacting to minor fluctuations in the training data. Moreover, large signatures are usually functionally redundant with less clinical utility and validation feasibility. The single-objective optimiser and penalised SVM produced very large signatures. On the other hand, Lasso produces models with no coefficient (with intercept only) in ~20% of runs.

We also estimated the stability of selected features across different runs using Jaccard Index that measures the intersection over union of two sets. Accordingly, Jaccard Index was computed for all pairs of signatures identified by each of compared methods across 50 runs and the average values were reported (Fig. 3c). The multi-objective optimiser exhibits signatures with significantly higher stability than those identified by the compared methods. Overall, the results demonstrate biomarker reproducibility using the proposed network-based multi objective optimisation approach.

### Identified plasma miRNA signature of CRC prognosis

Once we confirmed the predictive power and stability of the signatures obtained by the proposed multi-objective approach, we restricted the search space to miRNAs with clinically reasonable variations across samples with short vs long survival. This will assure that miRNAs contained in the final signature can be technically detected and verified in future experimental validations. We chose relatively loose yet clinically feasible fold-change > 1.5 in either directions (i.e., fold change computed as 2^∆∆Ct^ using ‘HTqPCR’ R package), which resulted in 51 miRNAs used to identify plasma signatures by the proposed multi-objective optimiser.

**Fig. 4:**
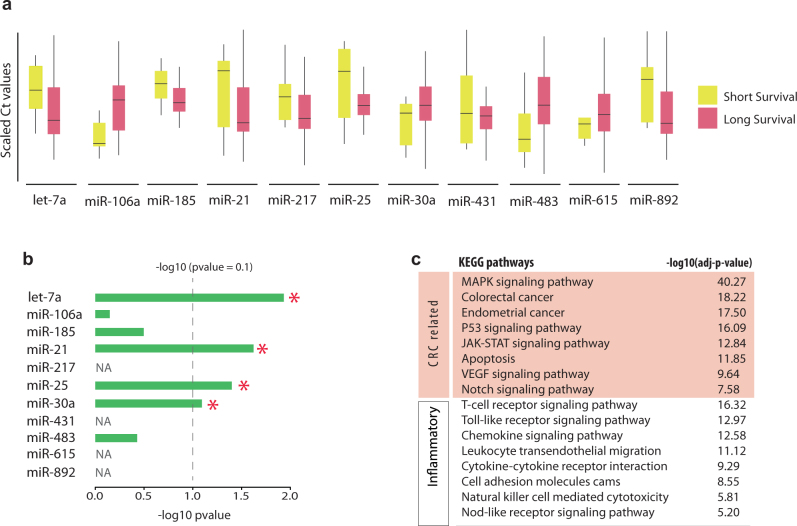
Identified plasma miRNA signature of CRC prognosis. A prognostic signature of 11 plasma miRNAs was identified using the proposed network-based multi-objective optimisation approach. a Boxplots represent the distributions of miRNA expressions across short and long survival samples. **b** The expression values of the identified miRNAs were examined in an independent public data set of qPCR miRNA profiles obtained from CRC plasma of patients at early or late cancer stages (accession no: GSE67075). Early-stage vs advanced cancer was compared using non-parametric Kolmogorov−Smirnov hypothesis testing. The bar in front of each miRNA shows the achieved *p* value scaled by –log10 to improve visibility. ‘NA’ indicates that the corresponding miRNA was not profiled (or filtered out) in the data set; ‘*’ specifies differentially expressed miRNAs based on the *p* value cut-off of 0.1. **c** List of important overrepresented KEGG pathways and their corresponding –log10 scaled *p* values, related to CRC mechanisms and inflammation that is an important risk factor for the development of colon cancer

We identified a prognostic signature (accuracy = 0.907, FR = 4.697) comprising 11 plasma miRNAs namely *hsa-let-7a*, *hsa-miR-106a*, *hsa-miR-185*, *hsa-miR-21*, *hsa-miR-217*, *hsa-miR-25*, *hsa-miR-30a-5p*, *hsa-miR-431*, *hsa-miR-483-5p*, *hsa-miR-615-5p*, *hsa-miR-892a1*. The statistical significance of the identified signature was assessed using a permutation test and a nominal *p* value of zero was achieved. Figure 4a shows boxplots representing the distributions of miRNA expressions across short and long survival samples. The expression levels of the identified miRNAs were examined in an independent public data set of qPCR miRNA profiles obtained from CRC *plasma* samples including eight early-stage and eight advanced samples.^55^ Four miRNAs in the identified signature were not profiled (or filtered out) in the plasma data set—i.e., *hsa-miR-217*, *hsa-miR-431*, *hsa-miR-615-5p* and *hsa-miR-892a*. Out of the seven remaining biomarker miRNAs, four miRNAs show significant differential expression based on the *p* value cut-off of 0.1 (Fig. 4b). This proportion is statistically significant (*p* value = 0.0084, Fisher’s exact test with parameters: *N* = 310, *K* = 72, *n* = 7, *k* = 4).

Targets of the identified miRNA signature enrich several cancer-related as well as inflammatory pathways. There is a well-established connection between inflammation and tumorigenesis with numerous supporting evidence from genetic, pharmacological and epidemiological data.^68^ Inflammation is an important risk factor for the development of colon cancer.^69^ Figure 4c shows some of the important pathways related to CRC mechanisms and inflammation that highlights the biological implications of the identified biomarkers in CRC development and progression.

Among the identified biomarker miRNAs, the utility of serum miRNA *miR-21*as a marker of CRC progression and diagnosis has previously been investigated.^15,16,55^ Downregulation of *miR-106a* in tumour was previously shown to predict shortened survival in patients with colon cancer.^70^ Also, experimental evidence suggests that the let-7 family contributes to immune evasion by the tumour and there is an association of *let-7a* expression with T-cell densities and mortality^71^ in CRC. STIM1, a direct target of *miR-185*, is associated with CRC poor prognosis and promotes tumour metastasis.^72^ *MiR-217* and *miR-25* in CRC tumours are associated with patient prognosis,^73,74^ and *miR-30a* has an inverse correlation with the staging in patients with colon cancer.^75^ *MiR-892a* was frequently upregulated in human CRC tissues and cell lines promoting cell proliferation and colony formation of CRC.^76^ Our study, however, is the first to demonstrate the utility of these miRNAs as *circulating* markers of CRC progression.

### Generality and flexibility of the proposed miRNA biomarker discovery approach

Although identifying circulating miRNA signatures of CRC survival was a major objective of the current study, the proposed network-based multi-objective approach is sufficiently general to identify signatures of disease phenotypes in other miRNA biomarker studies. To evaluate the generality and flexibility of the proposed approach, we sought for other miRNA biomarker discovery studies whose data sets are available to download from NCBI GEO repository.

Recently, circulating serum exosomal miRNAs have been studied as potential diagnostic markers of oesophageal adenocarcinoma.^77^ MiRNAs in serum exosomes were profiled from a cohort of 19 healthy controls and 18 individuals with locally advanced oesophageal adenocarcinoma using OpenArray real-time PCR platform. We downloaded the raw data (GEO accession no: GSE63108) and pre-processed using a similar pipeline followed in our study which resulted in 130 miRNAs for downstream analyses (see Supplementary file 1, Section 1.5 for preprocessing details). The corresponding miRNA-mediated gene regulatory network was then constructed and annotated by 33 genes associated with oesophageal adenocarcinoma in MalaCards (see Supplementary file 6 for the list of genes).

**Fig. 5:**
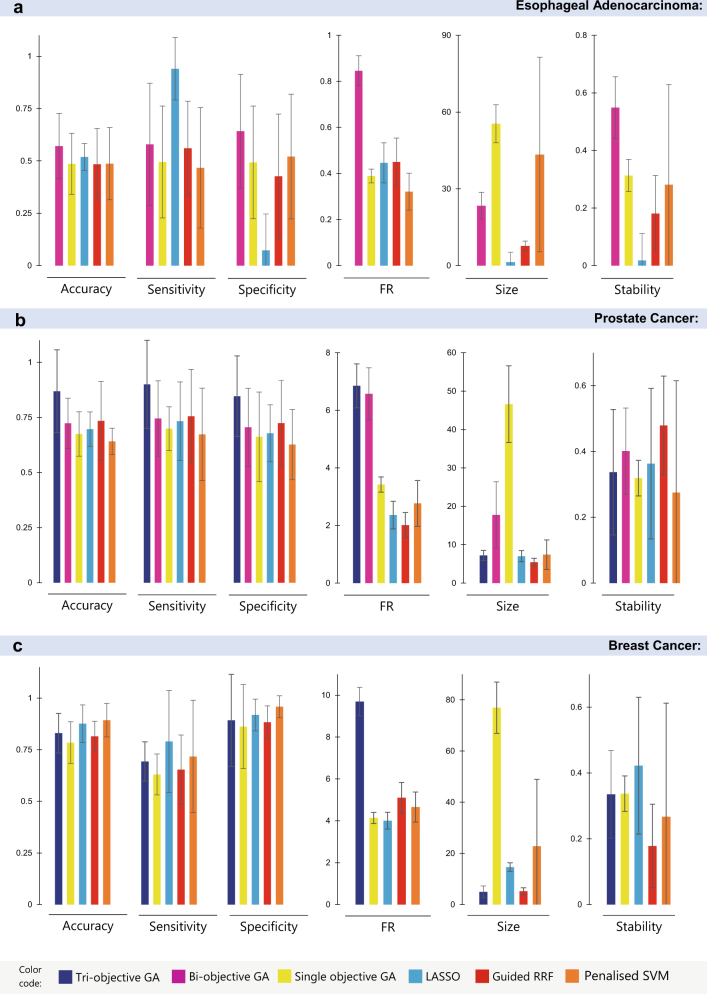
Performance comparison over three other miRNA data sets. The proposed multi-objective optimiser and four benchmark methods were used to identify signatures of disease phenotypes in three publicly available data sets. The performance measures (i.e., accuracy, sensitivity and specificity over test samples, functional relevance (FR), signature size and stability based on Jaccard index) of compared methods were aggregated across 25 independent runs (five runs of fivefold CV). Bar charts represent the average values and error bars show standard deviations. **a** GSE63108: circulating exosomal miRNA expression profiles in oesophageal adenocarcinoma and normal samples. **b** GSE76260: miRNA expression profiling in prostate cancer tumours vs non-neoplastic tissues. Bi-objective GA searches for signatures that simultaneously minimise the error rates and the inverse of FR. Tri-objective GA minimises error rate, 1/FR and signature size simultaneously. Increasing the number of objectives increases the number of Pareto front solutions. In tri-objective GA, a Pareto front solution performing better with respect to the first objective has been chosen in each run. **c** GSE70754: miRNA expression profiles in locally advanced breast cancer tumour vs normal tissues

We adopted a similar GA experimental setup used in previous experiments (i.e., population size of 100 and maximum number of 50 generations). Similarly, samples were divided into discovery and validation sets using fivefold cross-validation and repeated five times. In each run, compared methods (i.e., multi-objective optimiser, single-objective optimiser, Lasso, guided RRF and penalised SVM) were trained on the discovery sets and used to predict the validation samples. Average accuracy, sensitivity, specificity, functional relevance, signature size and signature stability were then reported for each of the compared algorithms. Results presented in Fig. 5a (and detailed in Supplementary file 1, Table S1) show that the bi-objective GA produced signatures with superior predictive power and higher relevance to the disease underlying mechanisms. Bi-objective GA feature selection was more robust to data partitioning and produced reasonably sized signatures with higher stability.

The optimisation-based biomarker discovery method is open to further enhancement by improving the adopted search mechanism. For instance, the search coverage can be extended by simply increasing the population size. We increased the population size to 200 and observed a better performance of single-objective GA while the performance of bi-objective GA was not significantly improved. We hypothesised that the early convergence of the bi-objective GA performance can be attributed to the miRNA network poor annotation due to the relatively small number of genes known to be associated to the disease under study. This limitation may direct the search algorithm towards the selection of fewer number of miRNAs resulting in immature convergence to local minima.

Therefore, to better assess the performance of the proposed method, we sought for relevant data sets on diseases whose associated genes are well studied and annotated in MalaCards producing relatively rich annotation on miRNA-mediated network. We retrieved miRNA expression profiles acquired from prostate clinical specimens, including 32 cancer and 32 non-neoplastic tissues^78^ (GEO accession no: GSE76260). Data were preprocessed as detailed in Supplementary file 1, Section 1.5, resulting in 103 miRNAs for the subsequent analyses. The corresponding miRNA-mediated regulatory network was annotated with 261 genes (including 29 elite genes) associated with prostate cancer in MalaCards (Supplementary file 6). We kept the GA population size at 200 and ran similar experiments performed for the previous data set; results are presented in Fig. 5b and Supplementary Table S1. Bi-objective GA identified signatures with significantly higher functional relevance scores. In terms of the predictive power, bi-objective GA exhibited performance comparable to guided-RRF (Wilcoxon *p* value = 0.861 comparing accuracies) and better performance compared to other methods. Guided-RRF however produced more compact signatures composing of fewer numbers of miRNAs.

To produce more compact signatures using the proposed multi-objective approach, we considered size to be the third objective resulting in a tri-objective GA search for miRNA signatures that simultaneously minimise the misclassification error rate, maximise the functional relevance and minimise the signature size. We also increased the population size and the maximum number of generations by 50% to achieve a more extensive search across the space of possible signatures and reran a fivefold cross validation. Average performance measures are reported in Fig. 5b. Interestingly, the tri-objective GA not only discovered small-sized signatures (average size = 7.2 ± 1.3), but also improved the predictive power by producing models with fewer number of variables which avoid overfitting to the training sets.

We acquired a third data set investigating miRNA diagnostic markers of breast cancer^79^ (GEO accession no: GSE70754). We retrieved normalised miRNA expression profiling of 66 samples including 19 normal specimens, from patients with locally advanced breast cancer during chemotherapy treatment. We preprocessed data as detailed in Supplementary file 1, Section 1.5 and ended up with 160 miRNAs used for biomarker discovery. As before, we constructed the miRNA-mediated regulatory network and annotated it with 317 genes (including 26 elite genes) associated with breast cancer by MalaCards (Supplementary file 6). We set the GA population size and maximum number of generations to 200 and 50, respectively. Signature size was retained as the third objective of the multi-objective optimisation approach. The performance measures of the compared methods were aggregated over five runs of fivefold cross validation (25 independent runs) and summarised in Fig. 5c and Supplementary Table S1. Results show that tri-objective GA outperforms its competitors with a higher functional relevance score and smaller signature size (average size = 5.0 ± 2.3) as it explicitly optimises for these objectives. It is the second best performing in terms of accuracy, sensitivity and specificity. Penalised SVM and Lasso produced signatures with higher predictive power but were larger in size.

## Conclusion

Accumulating evidence in recent years has convincingly demonstrated that the expression of various miRNAs is frequently dysregulated in CRC tissue.^80,81^ More importantly, recent studies have shown that some of these can also be detected in the circulation, and their expression pattern can be directly related to physiological and pathological alterations in patients with CRC.^8^ However, few circulating miRNAs so far have been reported as markers of CRC prognosis with limited consistency across different studies.^13–16^ In this study, we performed miRNA profiling using 75 plasma samples of locally advanced and/or metastatic colorectal patients. We identified a signature comprising 11 miRNAs with utility as biomarkers of CRC prognosis with significant alterations in an independent validation data set. The identified signature also corroborates previous findings on miRNA prognostic markers detected from plasma or tumours of CRC patients.

We have developed a powerful new miRNA biomarker discovery workflow to identify clinically and biologically relevant miRNA biomarkers by integrating advanced data-driven methodologies with a knowledge-based approach, utilising information from an miRNA-mediated network annotated with relevant cellular mechanisms. The miRNA-mediated regulatory network can exploit miRNA control in biological circuits, and provide insight into the consequences of miRNA dysfunction in disease. While miRNA direct targets have been increasingly studied in recent years and compiled in multiple data repositories, our study is the first to study a *network* of miRNA-mediated regulations representing deep regulatory cascades triggered by miRNAs. Such a network draws a more comprehensive picture of cellular regulations triggered by miRNAs as compared to miRNA-target direct interactions, and thus provides deeper insights into pathological phenomena associated with miRNA dysfunction. By constructing a network of miRNA-mediated regulatory cascades and incorporating measured data from this network into a multi-objective optimisation workflow, we have demonstrated the potential for data-driven, knowledge-based approaches to discovering new miRNA signatures. We have quantitatively compared the performance of our multi-objective approach to relevant approaches with inherent feature selection (e.g. single-objective optimiser, Lasso, guided RRF and penalised SVM) and demonstrated that our approach outperforms on all relevant metrics: accuracy, specificity, sensitivity, functional relevance and stability in this particular data set. We confirmed the generality and flexibility of the proposed method across three other publicly available data sets used to investigate miRNA diagnostic markers of oesophageal adenocarcinoma, prostate cancer and breast cancer. We demonstrated the advantage of using a data-independent knowledge base incorporated into a data-driven model to control overfitting to expression data and avoid producing excessively large signatures with poor predictive performance in independent data sets. Additionally, the multi-objective optimisation framework provides the flexibility to adjust for different objectives of interest and to incorporate heterogeneous yet relevant information facilitating systems approaches to biomarker discovery.

## Data and code availability

The circulating miRNA expression profile of CRC samples collected in this study can be accessed at NCBI Gene Expression Omnibus (GEO) using the accession number GSE112955. The proposed network-based multi-objective optimisation workflow for miRNA biomarker discovery was coded in MATLAB R2014b and R and is available at https://github.com/VafaeeLab/multiobj_miR_marker_discovery.

## Electronic supplementary material

Supplementary file 6

Supplementary file 1

Supplementary file 2

Supplementary file 3

Supplementary file 4

Supplementary file 5

## Electronic supplementary material

**Supplementary Information** accompanies the paper on the *npj Systems Biology and Applications* website (10.1038/s41540-018-0056-1).
