# Supplementary material for: A data-driven, knowledge-based approach to biomarker discovery: application to circulating microRNA markers of colorectal cancer prognosis
Source: NPJ Syst Biol Appl. 2018 Jun 1;4:20. doi: 10.1038/s41540-018-0056-1 (PMC5981448; doi:10.1038/s41540-018-0056-1)
Supplement: Supplementary file 2 — Supplementary file 1 [file 41540_2018_56_MOESM2_ESM.docx]

Supplementary file 1

**A data-driven, knowledge-based approach to biomarker discovery: application to circulating microRNA markers of colorectal cancer prognosis**

Contents

[1 Methods 2](#_Toc501104847)

[1.1 RNA isolation and quality control 2](#_Toc501104848)

[1.2 OpenArray analysis 2](#_Toc501104849)

[1.3 Data preprocessing parametrisation 3](#_Toc501104850)

[1.4 Construction of miRNA-mediated gene regulatory network 4](#_Toc501104851)

[1.5 Pre-processing details of datasets used to investigate generality of the proposed approach 5](#_Toc501104852)

[2 Results 6](#_Toc501104853)

[2.1 Visualization of Raw qPCR Data 6](#_Toc501104854)

[2.2 Histogram of FR scores 7](#_Toc501104855)

[2.3 Performance comparison on test and training samples 8](#_Toc501104856)

[2.4 Performance comparison across other miRNA datasets 9](#_Toc501104857)

# Methods

## RNA isolation and quality control

Total RNA was isolated from plasma using the MirVana PARIS microRNA isolation kit (Ambion/Applied Biosystems, Foster City, CA) according to the manufacturer’s instructions for isolation of total RNA, with modifications as described previously [*Kirschner et al. PLoS ONE 2011*]. RNA samples were stored at -80°C until further use.

Isolated plasma samples were assessed for haemolysis by examination of free haemoglobin and miR-16 levels, the latter being a miRNA found in red blood cells. Quantification of free haemoglobin was performed as described previously on an Implen Nanophotometer (Implen GmbH, Munich, Germany), measuring the absorbance of free haemoglobin at 414, 540 and 570nm [*Kirschner et al. PLoS ONE 2011*]. Samples with haemoglobin levels corresponding to an A414 reading of >0.25 and miR-16 Cq <19 or >21.5 were excluded from further analysis.

MiR-16 levels were quantified by real-time RT-qPCR. Reverse transcription was firstly performed using a LabNet MultiGene Gradient (Edison, NJ, USA) with the following reaction conditions: 30min at 16°C, 30min at 42°C, 5min at 85°C. The reaction was carried out with a total reaction volume of 5μl with a fixed volume of 1.67μl isolated RNA as the template. The resultant complementary DNA was used immediately in qPCR reactions.

After reverse transcription, the complementary DNA was diluted by addition of 28.9μl H2O so as to achieve a final dilution of 1:15 of the RT product [*Mitchell et al. P. Natl. Acad. Sci. U.S.A. 2008*]. 2.25μl of the diluted RT product was used as a template in triplicate qPCR with a total reaction volume of 10μl. Amplification was performed using SYBR Green primers specific for miR-16, and SYBR Green 2x MasterMix with enzyme activation for 10 minutes at 95°C, followed by 40 cycles of 15 sec at 95°C and 60 sec at 60°C. The qPCR reactions were set up manually on a Stratagene Mx3000P instrument, with Cq (quantification cycle) values determined using adaptive-baseline and background-threshold using MxPro Mx3000P v4.10 software (Stratagene/Agilent Technologies, Santa Clara, CA, USA). Cq values in the range of 19-22 were considered acceptable for proceeding.

## OpenArray analysis

Global profiling of miRNAs in the plasma samples was carried out using the BioTrove OpenArray platform (Applied Biosystems), according to the manufacturer’s instructions. For each sample, two RT reactions were performed using MegaPlex RT primer pools A and B (Applied Biosystems) with 100ng RNA per pool, on a LabNet Multigene Gradient with 40 cycles of 2 minutes at 16°C, 1 minute at 42°C, and 1 second at 50°C, followed by 5 minutes at 85°C. The entire RT reaction was used for preamplification. Preamplification was carried out on a ViiA 7 instrument (Applied Biosystems) using MegaPlex RT preamplification primer Pools A and B and MegaPlex preamp Master Mix (Applied Biosystems) under the following conditions: 10 minutes at 95°C, 2 minutes at 55°C, 2 minutes at 72°C, followed by 16 cycles of 15 seconds at 95°C, and 4 minutes at 60°C. Preamplification reactions were then incubated at 99.9°C for 10 minutes and cooled to 4°C. The resultant cDNA was diluted, and this frozen at -20°C until further use. The diluted cDNA was thawed, combined with the OpenArray real-time PCR Master Mix and loaded onto the OpenArray miRNA panel plates (Applied Biosystems) using the AccuFill autoloader. The loaded plates were run on the BioTrove OpenArray realtime PCR instrument (Flinders Medical Centre, SA) and run according to the default protocol for reaction conditions.

## Data preprocessing parametrisation

The preprocessing parameters have been carefully chosen in this study as discussed below:

**Filtering miRNAs**: Real-time qPCR data preprocessing workflow usually includes the estimation of non-detects [PMC4133581]. Imputation algorithms typically require the majority of miRNAs to have no missing values across samples in order to accurately estimate fewer miRNAs whose values are missed across more samples (see examples provided in [PMC4133581, reference to R package]). However, our qPCR data contains several missing miRNAs across multiple samples. Figure S1 shows the histogram of miRNA missing values. We chose the cut-off of 35 samples (i.e., 50%) to acquire the desired distribution for the downstream analysis. Overall, 150 miRNAs were included for the subsequent biomarker identification process.


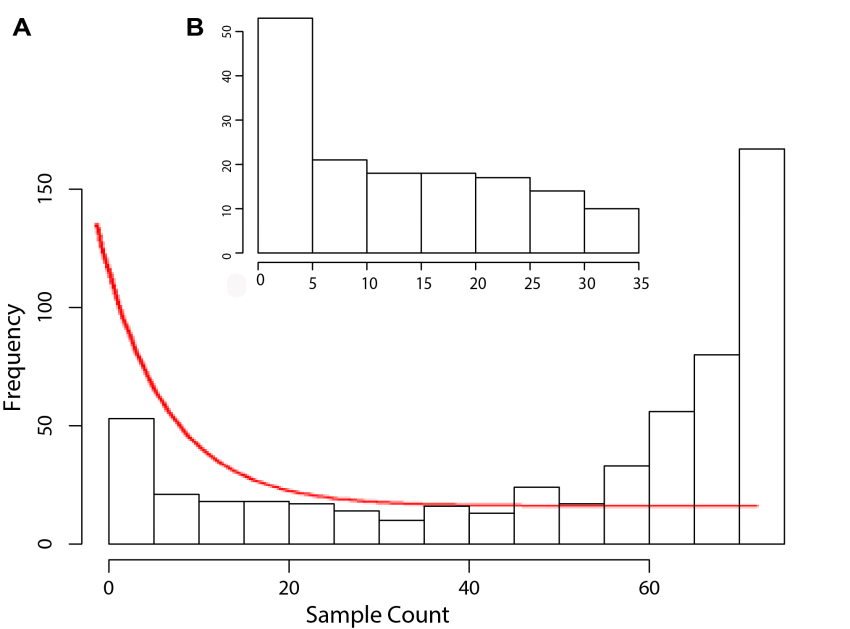


**Figure S1: Histogram of miRNAs missing values across samples. A) Before filtering; red curve indicates the desired distribution. B) Histogram after filtering.**

**Filtering samples**: Filtering was applied on samples after miRNA filtering. No sample was excluded in this step. We however preferred to mention the filtering undertaken to assure readers of the quality of the included samples.

**Classifier selection**: The proposed approach is classifier-independent. It provides a framework to automatically select features based on multiple criteria using a pre-selected predictive model/classifier. We tried different classifiers namely SVM, Random Forest, and AdaBoost (with decision trees as weak learners). SVM outperformed other classifiers when predicting the ‘short survival’ class which has less number of observations. We therefore chose SVM as the classifier for the optimisation process. SVM hyper-parameters were optimised automatically using MATLAB fitcsvm relevant arguments which attempt to minimize the cross-validation loss by varying the parameters. Nonetheless, the choice of parameters did not significantly affect the performance. The linear kernel function was chosen as it is substantially faster than polynomial kernel without affecting the performance.

**Survival dichotomisation**: We chose a 2-year cut-off to dichotomise patients’ survival as this is the best choice around the median/mean providing the highest marginal difference between short vs long survival. It is also a recommended cut-off by our clinicians. Figure S2 shows the survival values around median/mean (median = 21.23 and mean = 20.98 months) and clarifies that a 2 year (24 month) cut-off corresponds to the largest gap between short and long survival groups. Please note that we discretised the survival values rather than using continuous survivals due to the prevalence of censored values.

**Figure S2: Survival values around mean/median; dashed line shows the mean resulting in 4.7 month gap between short vs long survival group. Changing this cut off to 2 years increases the gap to 0.66 months.**

## Construction of miRNA-mediated gene regulatory network

**Input:**

- Set of miRNA-Gene pairs denoted by $S$
- Gene regulatory network represented as $G=(V,E)$where $V$ is the set of all TFs and TGs in ORTI and $E=\{(v,u)\}$corresponds to directed edges from T$F v$ to T$G u$

**Output:**

- miRNA-driven Regulatory Network denoted as $miRNet$

**Procedure** $BuildNet (G,S)$

1. add all miRNA-Gene pairs to $miRN$
2. **for** each Gene $g$**do**
3. run $AddEdges \left( G,g \right)$

**Procedure** $AddEdges (G,v)$

1. label $v$ as visited
2. **for** all $u$ such that $(v,u)\in E$**do**
3. **if** gene $u$ is not labeled as visited **then**
4. add $(v,u)$ to $miRNet$
5. recursively call $AddEdges (G,u)$

Figure S3. Recursive implementation of miRNA mediated gene regulatory network

## Pre-processing details of datasets used to investigate generality of the proposed approach

**GSE63108: circulating serum miRNAs as diagnostic biomarkers for *esophageal adenocarcinoma* [PMID: 25943911]**

Similar to our study, this work used OpenArray real-time PCR platform to profile 758 miRNAs in serum circulating exosomes from a cohort of 19 healthy controls and 18 individuals with locally advanced esophageal adenocarcinoma. MiRNA identifiers were standardised against miRBase-version 21 using miRSystem online tool^[[1]](#footnote-1)^; unknown or dead miRNAs were excluded. MiRNAs missing in > 50% of samples as well invariant miRNAs (IQR < 1.5) were filtered out resulted in 130 miRNAs for downstream analysis. Data was normalised using quantile normalization and missing values were imputed using the nearest-neighbour method (KNNimpute).

**GSE76260: microRNA expression profiling in *prostate cancer* tumours versus non-neoplastic tissues [PMID: 28274892**]

In this work, miRNA expression profiles in a series of 64 prostate clinical specimens, including 32 cancerous and 32 non-neoplastic tissues were assayed using an Illumina platform targeting 1,146 miRNAs. The preprocessd data (i.e., log2-transformed and normalized by the robust spline method using Bioconductor package ‘lumi’) were retrieved from GEO. MiRNA identifiers were standardized to miRbase-version 21using miRSystem. Non-human and invariant miRNAs were filtered out; 103 miRNAs were remained for subsequent analyses. Imputation was not required for this platform.

**GSE70754: microRNA expression in locally advanced *breast cancer* tumour versus normal tissues [PMID: 27064979]**

Affymetrix Multispecies miRNA-2 Array has been used in this work to profile miRNA expressions in locally advanced breast cancer tumours and normal tissues (66 specimens including 19 normal samples). The preprocessed data (i.e., log2-transformed and normalized by RMA-DABG method using in Affymetrix ExpressionConsole) were downloaded from GEO. MiRNA identifiers were standardized to miRbase-version 21using miRSystem online tool and non-human miRNAs were excluded. 160 miRNAs whose |logFC (cancer *vs* normal)| ≥ log2(1.5) were considered for the subsequent biomarker discovery. Imputation was not required for this platform.

# Results

## Visualization of Raw qPCR Data


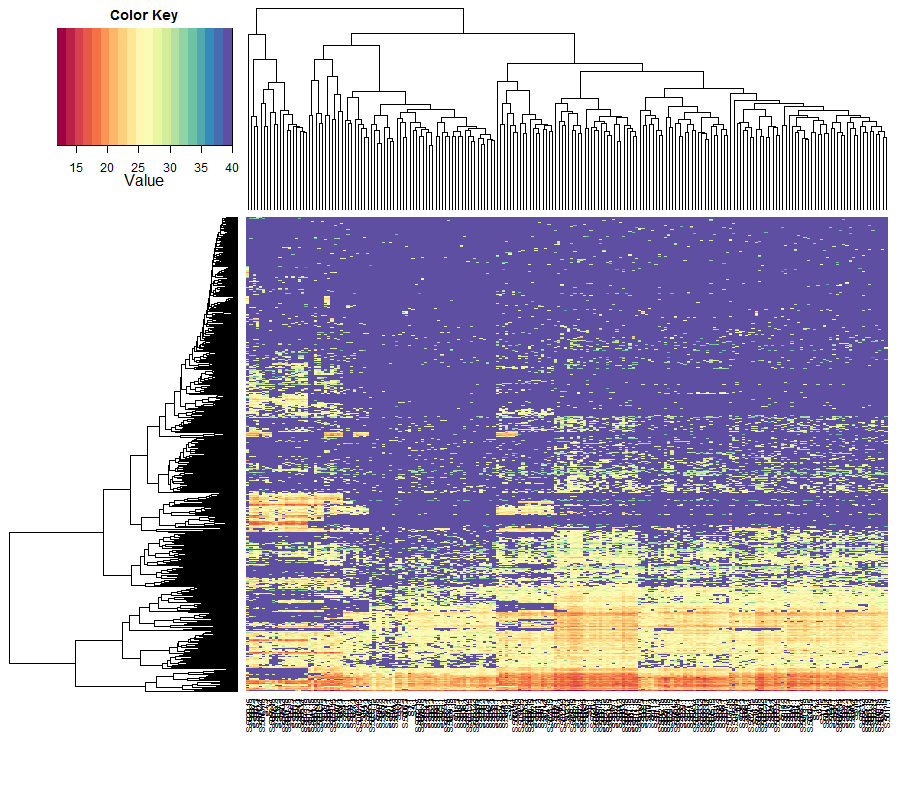


Figure S3. Histogram of Ct values, 564 miRNAs (rows) over 75 samples (columns). Ct value of 40 (purple dots) corresponds missing values.

## Histogram of FR scores


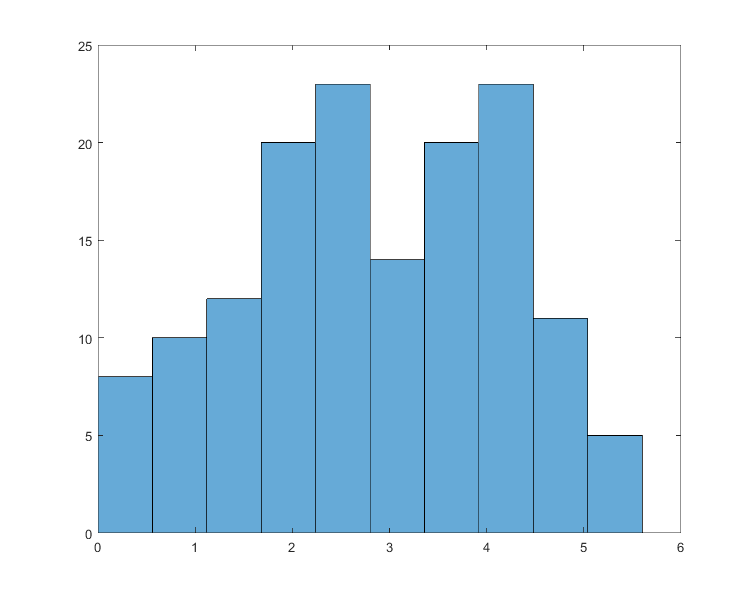


**Frequency**

**FR score**

Figure S4. Histogram of miRNAs’ FR scores

## Performance comparison on test and training samples

Performance on **test** samples

Performance on **training** samples

Multi-objective Optimisation

Single-objective Optimisation

Figure S5. Performance of multi- vs single-objective optimisers on training and test sample; bar charts show average values over 50 cross-validation runs.

## Performance comparison across other miRNA datasets

Table S1 Three publicly available datasets (i.e. GSE63108, GSE76260, and GSE70754) were used to identify diagnostic miRNA signatures in other diseases using 5 comparing methods. The performance measures of compared algorithms were aggregated across 25 independent runs (5 runs of 5-fold CV). Average values and standard deviations (in parenthesis) were reported.

|  |  | | Accuracy | | Sensitivity | | Specificity | | FR | | Size | | Stability | |  |
| --- | --- | --- | --- | --- | --- | --- | --- | --- | --- | --- | --- | --- | --- | --- | --- |
| Oesophageal | | Bi-Obj GA | | 0.571 (0.156) | | 0.579 (0.292) | | 0.642 (0.272) | | 0.846 (0.065) | | 23.417 (5.238) | | 0.549 (0.107) | |
|  |  | Single Obj GA | | 0.486 (0.146) | | 0.495 (0.267) | | 0.493 (0.269) | | 0.389 (0.03) | | 55.44 (7.428) | | 0.313 (0.056) | |
|  |  | LASSO | | 0.519 (0.064) | | 0.94 (0.149) | | 0.073 (0.174) | | 0.446 (0.087) | | 1.36 (3.872) | | 0.018 (0.094) | |
|  |  | Guided RRF | | 0.484 (0.171) | | 0.56 (0.225) | | 0.427 (0.297) | | 0.45 (0.104) | | 7.72 (1.882) | | 0.181 (0.132) | |
|  |  | Penalised SVM | | 0.487 (0.172) | | 0.467 (0.288) | | 0.521 (0.298) | | 0.321 (0.08) | | 43.4 (38.005) | | 0.281 (0.348) | |
| Prostate Cancer | | Bi-Obj GA | | 0.723 (0.114) | | 0.745 (0.171) | | 0.705 (0.177) | | 6.575 (0.905) | | 17.76 (8.647) | | 0.401 (0.131) | |
|  |  | Single Obj GA | | 0.675 (0.101) | | 0.699 (0.099) | | 0.662 (0.203) | | 3.421 (0.264) | | 46.6 (5.331) | | 0.319 (0.054) | |
|  |  | LASSO | | 0.697 (0.078) | | 0.733 (0.178) | | 0.678 (0.129) | | 2.361 (0.482) | | 7 (1.443) | | 0.363 (0.229) | |
|  |  | Guided RRF | | 0.734 (0.179) | | 0.755 (0.213) | | 0.724 (0.194) | | 2.012 (0.437) | | 5.44 (1.003) | | 0.479 (0.15) | |
|  |  | Penalised SVM | | 0.641 (0.06) | | 0.673 (0.21) | | 0.627 (0.159) | | 2.766 (0.794) | | 7.4 (3.851) | | 0.275 (0.34) | |
| Breast Cancer | | Tri-Obj GA | | 0.869 (0.188) | | 0.9 (0.2) | | 0.846 (0.183) | | 6.85 (0.763) | | 7.2 (1.304) | | 0.337 (0.191) | |
|  |  | Tri-Obj GA | | 0.83 (0.096) | | 0.892 (0.095) | | 0.693 (0.223) | | 9.694 (0.681) | | 4.96 (2.336) | | 0.335 (0.133) | |
|  |  | Single Obj GA | | 0.784 (0.101) | | 0.862 (0.099) | | 0.63 (0.203) | | 4.135 (0.264) | | 76.92 (5.331) | | 0.337 (0.054) | |
|  |  | LASSO | | 0.876 (0.091) | | 0.79 (0.247) | | 0.918 (0.077) | | 4.007 (0.397) | | 14.64 (1.68) | | 0.422 (0.208) | |
|  |  | Guided RRF | | 0.815 (0.073) | | 0.653 (0.168) | | 0.883 (0.079) | | 5.1 (0.729) | | 5.24 (1.332) | | 0.178 (0.127) | |
|  |  | Penalised SVM | | 0.893 (0.081) | | 0.717 (0.272) | | 0.958 (0.053) | | 4.658 (0.715) | | 22.8 (26.152) | | 0.267 (0.345) | |

1. <http://mirsystem.cgm.ntu.edu.tw/index.php> [↑](#footnote-ref-1)
